# Controlling Prescribing through “Preferred Drug” Targets—The Bavarian Experience

**DOI:** 10.3390/ijerph21091174

**Published:** 2024-09-03

**Authors:** Julia Gollnick, Nikoletta Zeschick, Julia Muth, Franziska Hörbrand, Kerstin Behnke, Peter Killian, Maria Sebastiao, Thomas Kühlein, Norbert Donner-Banzhoff

**Affiliations:** 1 Institute of General Practice/Family Medicine, Philipps University of Marburg, Karl-von-Frisch-Straße 4, 35043 Marburg, Germanynorbert@staff.uni-marburg.de (N.D.-B.); 2 Institute of General Practice, Friedrich-Alexander-University of Erlangen-Nürnberg (FAU), Universitätsstr. 29, 91054 Erlangen, Germanymaria.sebastiao@uk-erlangen.de (M.S.); thomas.kuehlein@uk-erlangen.de (T.K.); 3 Association of Statutory Health Insurance Physicians, Bavaria, Elsenheimerstraße 39, 80687 München, Germany

**Keywords:** drug prescriptions, ambulatory care/economics, budgets, drug therapy/economics, drug therapy/trends, cost control/trends

## Abstract

Background: The rising costs of drugs are putting health care systems under pressure. We report on the Bavarian Drug Agreement, which employs prescribing targets for preferred and generic drugs in ambulatory care. Under this agreement, providers are regularly profiled with individual feedback but also possible sanctions. We investigated the degree to which targets were being met (or not) and why failure occurred. Methods: We analysed prescribing data aggregated by practice for the quarter 1/2018. We chose eight specialisation groups and analysed their drug targets with a high prescribing volume, widely missed drug targets (<90%), and drugs preventing drug target achievement. Characterisation of drug targets and preventing drugs was undertaken. Results: Drug targets with a high prescribing volume are mostly achieved, while highly missed drug targets mostly do not affect the main indication area of the specialisation groups considered. Generic drug targets seem to be more easily achieved than recommended drug targets. Paediatrics accounts for the largest number of missed drug targets. Conclusions: The Bavarian tool implemented uses the prescribing volume (DDD) and price components to evaluate the prescription behaviour of physicians. Well-established drugs with demonstrated effectiveness, safety, and lower costs are preferred. Nevertheless, me-too drugs, combination drugs, costly innovations with unclear value, and drugs with application methods of variable convenience challenge the drug prescribers and are reasons for missed drug targets.

## 1. Background

The rising costs of medicines are posing an increasing challenge for the health care system. Within the German health care system, federal law requires physicians to consider efficiency when prescribing drugs. Prescriptions must be clinically adequate, but the cost must be considered, and waste has to be avoided (§12 SGB V: Sozialgesetzbuch, which is the compilation of federal laws pertaining to health care). To monitor compliance with the efficiency requirement in the ambulatory sector, various surveillance instruments have been developed and introduced in a cooperation between statutory health insurance (SHI) funds and associations of SHI-accredited physicians (KVs) (§106b SGB V).

Surveillance procedures are needed because all drugs marketed within the European Union must be covered by SHI [1]. While coverage in most health care systems is restricted to those who have undergone not only clinical but also economic appraisal, in the German health care system, this decision is the responsibility of individual prescribers [2,3].

Data on drugs prescribed for SHI members are collected, stored centrally, and thus available for the profiling of practices regarding their prescribing. At this time, there are no electronic prescriptions available in Germany, so printed prescriptions are processed by pharmacies’ data centre. These data are made available to the SHI and KV for payments and prescription analyses.

If prescribers are not compliant with the efficiency requirement, the additional costs caused can be recovered on behalf of the SHI. Although this happens only in very rare cases, prescribers often express feelings of being threatened by sanctions of this kind [4,5]. The regulations apply to ambulatory care only and not to drugs administered by hospitals.

The established procedures focus mainly on surveying the prescribing costs per patient. A novel drug agreement was introduced in Bavaria (Bavarian Drug Agreement (BDA)) in 2014, which, instead of costs, was based on preferred drug targets measured in defined daily doses (DDD) [6].

A total of 32 drug targets were defined for 31 indication areas. Targets imply either a predefined percentage of generic prescriptions or a percentage of recommended drugs, referring to a particular class of drugs. In the BDA, eight recommended drug targets and 23 percentage generic targets are defined. For example, 91.5% of all antiepileptic drugs that neurologists prescribe must be generic drugs, and family doctors must prescribe at least 53.0% vitamin K antagonists (VKAs) as recommended drugs among oral anticoagulants (VKAs and DOACs). While the kinds of drug targets are identical for all specialities, the percentages required for meeting a target differ from one specialisation group to another. Recommended drugs are selected on the basis of clinical practice guidelines and assessment by the Federal Joint Committee (G-BA), which is the central regulating body of the German health care system.

Although the same drug targets apply to all the specialisation groups, some targets concern the core of a speciality while others are peripheral to their prescribing practice. While oral coagulants for atrial fibrillation are frequently prescribed by cardiologists and primary care physicians, neurologists rarely prescribe these. For this reason, drug targets are enforced only if a minimum prescribing volume measured in DDD occurs [7].

The drug target “proton pump inhibitors” (PPIs) was introduced to prevent extensive prescribing of PPIs. The target is reached if the prescribed PPI volume per patient remains below a defined level, for example 21.70 DDD for primary care or 1 DDD for neurologists [6,7,8]. The denominator is any patient who has obtained a drug prescription. Given the large variety of indications for PPI, prescriptions of PPIs are not analysed with regard to particular condition (of several), but to all patients who have received a prescription (any). In theory, a general practitioner could prescribe 21.70 DDD PPI for every patient who receives a prescription from them (regardless of which drug). In practice, these will be distributed among a limited number of patients, for whom PPIs are (or seem to be) indicated.

Practices obtain regular feedback regarding missed or reached targets. Each quarter they receive an evaluation on how well drug targets have been achieved and which drug prescriptions oppose achievement of drug targets. A cumulative overall target achievement is calculated; this includes the individual drug targets of the practice with a prescribed minimum measured quantity of DDD [7] weighted by an efficiency factor and the prescribing volume measured in DDD. In cases where more than the required percentage of preferred drugs are prescribed, the overall target achievement can be higher than 100%. Missing a target in one indication area can be compensated for by meeting a target by a large margin in another indication area. For all drug targets, practices receive detailed information on prescribed drugs that have led an individual practice to miss a particular target.

At the time of the study, the members of a speciality group can compensate for each other’s prescribing behaviour. If the speciality meets a particular target as a whole, individual practices and practitioners will be neither investigated nor sanctioned.

Within the German health care system, internists provide either primary care or specialised care. In the following, we treat general practitioners (GPs) and primary care internists together as “primary care”. The remaining internists are either subspecialists, for example cardiologists, or general internists without subspecialisation (in the following, “general internists”). Paediatrics includes primary care paediatrics as well as specialists. The latter must meet the same drug targets and percentage of generic and recommended drugs as their primary care colleagues.

Within the framework of the project *WirtMed—The Prescription of Medicines: Audit and Control of Efficiency and Quality*, funded by the Innovation Fund of G-BA, various audit and control procedures, as well as prescription patterns of drugs, are investigated. In the subproject reported here, we sought to investigate the effect of the prescribing targets specified within the BDA. In particular, we addressed the following research questions:

How many drug targets are relevant for each specialisation group with regard to their prescribing volume and typical indication area?

Which are the three drug targets with the highest prescribing volume, measured in DDD, in each specialisation group?

Which drug targets are typically missed? Which of these are generic targets, recommended drug targets, or PPI targets?

Are there particular drugs which prevent the achievement of high-volume drug targets?

## 2. Methods

Analyses were carried out using routine data from the Bavarian KV. Prescriptions are processed in the pharmaceutical data centre and automatically read, and the data are transferred into a database. Every quarter, the data are made available to the KV and SHI. The KV monitors efficiency requirements in the ambulatory sector and gives feedback to the physicians. The data kept by the KV include information accumulated from 25 specialisation groups, which represent all the practices in the ambulatory sector for the time period quarter 2/2017 until quarter 1/2018. For this purpose, two anonymised datasets on prescription data were provided to the Philipps-Universität Marburg.

Our first dataset contained monitoring data on compliance with the efficiency requirements. Therefore, the KV aggregated prescription data and submitted information about the prescribed DDD volume for every drug target in all the individual specialisation groups. Furthermore, for all the individual specialisation groups, they calculated the percentage of the prescribed DDD volume of preferred drugs in the prescribed DDD volume of all the prescribed drugs within a drug target and submitted the percentage of compliance with the drug targets for every drug target within a specialisation group. Thus, we received detailed information about the prescribed DDD volume and drug target achievement of every specialisation group.

We regard drug targets that include a high prescription volume measured in DDD and reflect the typical indication area of a specialisation group as “relevant”. For example, the drug target “drugs for the treatment of bone diseases” is a relevant drug target for orthopaedists and “ophthalmics” is a relevant drug target for ophthalmologists.

Our second dataset comprised the specific drugs per quarter and the particular amount prescribed for individual specialisation groups that prevented the target achievement for quarters 2/2017 to 1/2018, measured in prescribed DDD volume. We used the categorisations for drugs employed by the Bavarian KV (see Supplementary Materials Table S1).

The data for quarter 1 in 2018 were analysed and the two datasets were matched. We chose the first quarter of 2018 because it was the most recent.

The first analysis was a comparison of the number of relevant drug targets of the various specialisation groups (“relevant” as defined above). For more detailed analyses, we selected eight specialisation groups (Table 1) because of their high prescribing volume (e.g., general practitioners, general internists), treatment management of diseases with high cost drugs (e.g., neurology—MS; gastroenterology—IBD), and paediatrics, which includes primary care for children and specialty care consisting of high-volume prescribers and/or including a large number of practices that are not meeting their targets. The choice was discussed with the project-team, who included physicians, pharmacists and members of the KV, which developed the BDA.

For these eight specialisation groups, we identified the three drug targets accounting for the highest prescription volume, measured in DDDs, and considered whether they had been missed (drug target achievement <100%) or not (drug target achievement =/>100%). Furthermore, we looked at all the drug targets that were widely missed by a large margin (drug target achievement <90%). To estimate the relevance of the widely missed drug targets, the prescribed DDD volume was evaluated as well.

All the identified drug targets (highest prescribing volume and highly missed targets) were further characterised according to their type of target (percentage generic, percentage recommended drug, PPI) and how well the drug targets were achieved measured as a percentage. For all drug targets, we identified three drugs that were notorious for preventing target achievement.

**Figure 1:**
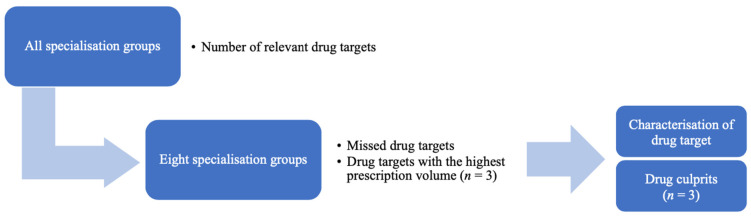
Steps of the data analysis and questions.

The following flow chart (Figure 1) shows the different levels of observation carried out of the prior level and the researched questions.

Analyses were carried out with ExCel.

We obtained permission from the Bavarian government for our work (SGB I, §287 Abs. 1). Approval from the Ethics Commission of the Faculty of Medicine was not required because we analysed only aggregated data extracted from the KVB database, which serves administrative purposes. Aggregate data were provided to the Department of General Practice and Family Medicine (Philipps-University Marburg) in ExCel format.

## 3. Results

### 3.1. Number of Relevant Drug Targets

The relevant drug targets, as specified above differ between specialisation groups, ranging from two (ophthalmologists) over eight (gastroenterology) to 23 (primary care) as the specialisation group with the highest number. For more detailed information on the different numbers and kinds of relevant drug targets of all 25 specialisation groups, see Table S2 in the Supplementary Materials.

### 3.2. Three Drug Targets with the Largest Prescription Volume within a Specialisation Group

In Table 2, we report the three drug targets with the highest prescription volume independent of target fulfilment for each of the eight defined specialisation groups. Further drug targets with a high prescribing volume might occur and be relevant to the overall target achievement within these specialisation groups, but, in this survey, we focused on the three drug targets with the highest prescribing volume.

The drug targets with the largest volume of prescriptions are predominantly generic (*n* = 19) followed by PPI target (*n* = 4) and recommended drug targets (*n* = 1). In most specialisation groups, the three drug targets with the highest prescribing volume are achieved (19 of 24 targets; target achievement >100% within a specialisation group). The exceptions are the specialisation group of neurologists, who miss two of the three drug targets (“other psychotropic drugs” and “antiepileptics”), and the specialisation groups of general internists (“combined group cardiovascular system”), paediatrics (“agents for obstructive respiratory diseases”), and cardiologists (“lipid-lowering agents”), each of which miss one drug target. All the missed high-volume drug targets within these eight specialisation groups are generic targets. All the high-volume drug targets show more than 90% target achievement; in other words, they were missed by a tight margin.

To understand why high-volume drug targets are missed, we identified the drugs preventing drug target achievement. The three drugs most frequently prescribed (DDD volume) by each specialisation group within the identified missed drug targets (Table 2) are shown in Table 3. For this purpose, preventing drugs were listed and ranked according to their prescribed DDD volume. The three top drugs were selected. For further information regarding the drugs check the Supplementary Materials Table S3.

In the group of general internists, the most problematic drugs have a share of less than 1 percent of the prescribed DDD volume within the drug target. Here the tendency to prescribe brand name drugs rather than generic ones leads to the missing of targets. For all the other drug targets considered, the problematic drugs have a higher impact (2.67 to 7 percent of the prescribed DDD volume). For the lipid-lowering class, all three drugs contain ezetimibe and are brand names. Single ezetimibe is regarded as inferior to statins. Besides prescribing leading brand names, the prescribing of pseudo-innovations, such as me-too drugs (Nebivolol (generic)) or new combinations of well-established drugs (Viani^®^), increases the risk of missing the drug targets. As a me-too drug, nebivolol is an expensive generic drug, which costs more than the defined fixed price and is therefore handled like a brand name.

### 3.3. Missed Drug Targets by Specialisation Group

Within the eight specialisation groups, we identified drug targets that showed <90% target achievement (Supplementary Materials Table S4). Among the missed drug targets, recommended drug targets (*n* = 16) are somewhat more frequent than generic drug targets (*n* = 13). Likewise, the missed targets are mainly targets with a low prescription volume measured in DDD. If one considers the indication related to missed drug targets, there are predominantly targets that do not belong to the core of drugs prescribed by a group, such as “antiparkinsonian drugs” prescribed by orthopaedists. Because of this, these failures to meet targets would not have consequences for prescribing practices.

The specialisation group of paediatrics has a comparatively high number of missed drug targets. At the same time, primary care misses only two drug targets, although it has the highest number of relevant drug targets (see above). Furthermore, the anticoagulants target is missed in almost every one of the eight specialisation groups analysed. Here the recommended drugs are vitamin K antagonists (VKA), while direct oral anticoagulants (DOACs) are increasingly prescribed.

### 3.4. Drugs Causing Targets to Be Missed

As well as the drugs in Table 3, we identified further drugs which prevent the achievement of high-volume drug targets even if they are achieved. These drugs usually have a markedly higher prescribing volume than other drugs preventing drug target achievement. Hence, the former drugs have a superior impact on the missing of targets: Voltaren^®^ (diclofenac), Exforge^®^ HCT (amlodipine, valsartan, hydrochlorothiazide), Prolia^®^ (denosumab), Humira^®^ (adalimumab), and Nurofen^®^ (ibuprofen).

In the field of orthopaedics, the three drugs that mostly prevented the drug target achievement of “drugs for the treatment of bone diseases” are all drugs to treat osteoporosis with parenteral application (Prolia^®^ (denosumab), Aclasta^®^ (zoledronate), Bonviva^®^ pre-filled syringe (ibandronate)) as opposed to oral preparations.

## 4. Discussion

Depending on the specialisation, the numbers of relevant drug targets show high variability. Missed drug targets are often peripheral to the prescribing repertoire of a specialisation group. Recommended drug targets are more frequently missed than generic ones. High-volume drug targets, however, are typically generic, and most of these are achieved. Some drugs and prescribing practices seem to be notorious for leading to missed targets in high prescribing areas.

### 4.1. Interpretation of Findings

#### 4.1.1. Definition and Justification of Targets

The goal of the BDA is to improve the prescribing practice regarding the cost, effectiveness, and safety of drugs. Generic drugs are favoured by the BDA not only because of their lower cost but also because they usually have a more reliable evidence base as the first drugs in their class. So called me-too drugs are often more expensive and lack a documented advantage over established drugs [9].

Me-too drugs and combined preparations of well-established drugs are often more expensive than single drugs. They are regarded as brand name drugs and thus lead to generic drug targets being missed. Recommended drugs overlap to a large degree with drugs favoured by clinical practice guidelines.

Drug targets will be made according to scientific evidence and the development of the drug market [10]. Since new medicines must first prove themselves against existing alternatives, it may take some time for them to be included in the drug targets.

Reaching a generic drug target is presumably easier because prescribers usually have more choice than in areas covered by a recommended drug target. At the same time, the number of recommended drug targets is smaller than those pertaining to generic targets. overall make up the smallest part of the drug targets. The most frequently defined generic targets also represent the main part of the targets with a high prescription volume within the considered targets and specialisation groups [7].

Although the choice of an established drug available in generic form and suggested by guidelines is usually adequate and rational, in selected cases, the characteristics of a patient may require alternative choices. The BDA accommodates this possibility by requiring not 100% fulfilment but, for example, 63% in drugs for the treatment of bone diseases by orthopaedists [11].

Overall, missed drug targets tend to be ones that only account for a small volume of prescriptions issued by that group and thus do not belong to the relevant drug targets (see the “methods” section above for the definition). As a result, they are not included in the feedback for each practice.

The rationale of defining the individual drug targets includes cost and effectiveness as well as safety. Within the drug target “lipid-lowering agents”, especially Ezetrol^®^ (7% of the prescribed DDD volume within this drug target) has an impact on the missing of the drug target. Ezetrol^®^ (ezetimibe), Inegy^®^ (ezetimbe, simvastatin), and Atozet^®^ (ezetimbe, atorvastatin) all include ezetimibe as an active drug. The patent for Ezetrol^®^ expired in May 2018 [12], while our considered data include quarter 1/2018. In this time period, a prescription of ezetimibe was not possible in a generic form. Additionally, ezetimibe is recommended in the case of statin-intolerant patients or in combination with statins to lower LDL values more effectively [13]. Ezetimibe was heavily marketed before studies concerning its effect on clinical endpoints became available. Even after the IMPROVE-IT study, ezetimibe must be regarded as a drug with weak and uncertain clinical relevance [14]. In Germany, single ezetimibe is not a recommended first-line therapy [15].

Due to the use of an algorithm for cumulating and weighting the individual drug targets of one practice within the overall target achievement, missing a single drug target does not automatically lead to a specialisation group missing the overall target achievement because compensation between targets is possible.

#### 4.1.2. Mechanism for Targets Being Missed

Specialisations with a large number of relevant drug targets must juggle multiple drug targets simultaneously. Contrary to expectations, this does not lead to more targets being missed. Primary care, which has the most relevant drug targets, missed only two drug targets.

The specialisation group of paediatrics has the largest number of missed targets among the specialisation groups. Here the question may arise of whether a system that was developed for adults can also be applied to children without further adaptation [7,8].

Looking at the missed drug targets within paediatrics, particularly drug targets related to severe, rare diseases are involved. For example, within the drug target “TNF-alpha inhibitors”, three of four recommended drugs include infliximab, which has no market authorisation for therapy of children under six years old and is recommended for children from six to 17 years only for chronic inflammatory bowel disease [16,17,18]. The treatment of younger children or children weighing less than 62.5 kg for juvenile idiopathic arthritis is only possible with biologicals, because of the available possibilities for individual weight-adapted dosages [19,20,21], but they are not recommended. A few months after our analyses, biosimilars of adalimumab were placed on the market, thus increasing the therapeutic options. The targets were modified accordingly.

Concerning those recommended drug targets that were missed, especially the target of anticoagulants seems to cause difficulties as this was missed by almost all of the eight specialisation groups considered. Regarding this drug target, the prescription of VKA was recommended, while DOACs should be prescribed only in selected cases. Our analysis included years when DOACs were available as brand names [22], and as other authors showed, marketing is predominantly related to brand names without generic availability [23]. Promotional efforts focused on hospitals where patients taking VKAs for long time periods were often switched to DOACs [24,25]. Afterwards, physicians in the ambulatory sector must prescribe these drugs further. DOACs show some advantages (simplicity, major bleeding), but also disadvantages (missing incompliant patients), and costs have to be kept in mind while prescribing these drugs (cost–benefit analysis), which is shown in the literature from 2010 and 2014 [26,27].

Further reasons for missing drug targets:

Way of application

Among the drugs for osteoporosis, denosumab (Prolia^®^) has by far the greatest influence on the failure to reach the drug target. In addition to the parenterally administered Prolia^®^, Aclasta^®^ (zolendronic acid), and Bonviva^®^ pre-filled syringe (ibandronic acid), parenteral application forms are in the second and third places among the drugs to be prescribed with lower priority. If oral bisphosphonates are to be avoided because of side effects or if there is a preference for parenteral application, prescribers risk the drug target being missed.

A drug that prevents the achievement of the drug target TNF-alpha inhibitors is adalimumab (Humira^®^). Adalimumab is available as a subcutaneous injection, which can be applicated at home by the patient, while other alternatives, such as infliximab, are administered as an intravenous injection in practice [28]. A preference for adalimumab therefore carries the risk of a target being missed (before biosimilars expanded the market).

Drugs with a narrow therapeutic range

For the drug target “antiepileptic drugs”, the two drugs that are most likely to prevent the achievement of the target are Orfiril^®^ (sodium valproate) and Ergenyl^®^ (sodium valproate, valproic acid). For valproic acid and its salt valproate, prescribers should not switch between preparations by different manufacturers because of the narrow therapeutic range and variability in bioavailability [29]. Conversely, prescribers are encouraged to change from brand to generic drugs. To initiate new therapies, drugs such as lamotrigine and levetirazetam are recommended, for which the problem of the substitution ban does not arise [30].

Pseudoinnovations

Physicians who want to be innovative and prescribe new drugs in some cases find themselves in trouble with the drug target achievement. Within the drug target “drugs with an effect on the RAS system”, especially the drug Exforge^®^ HCT (amlodipine, valsartan, hydrochlorothiazide) prevents drug target achievement. Prescribing combined preparations containing more than one drug is often suggested to reduce the number of tablets that patients have to take, but these brands are often overpriced and not cost-effective.

In relation to the drug target “combined group cardiovascular system”, the drugs nebivolol (generic), Nepresol^®^ (dihydralazine), and Carmen^®^ (lercanidipine) are the three drugs that mostly prevent the achievement of the generic drug target. Both nebivolol and lercanidipine are me-too drugs without a proven advantage but with a higher price. Nebivolol was supposed to have advantages over other beta-blockers, including vasodilatory effects via NO-dilution [31,32]. Nevertheless, the relevance of these mechanisms could not be shown in long-term randomized controlled studies [31].

### 4.2. Discussion of the Literature

Only a few studies have investigated compliance with the BDA [6,33]. Most of them have reported rising drug target achievement after the introduction of the BDA. Missed drug targets were beyond the focus of these studies. To our knowledge, no study has analysed the drug prescribing of different specialisation groups, described their missed drug targets and the drugs preventing the drug target achievement, and characterised the mechanism through which drug targets are missed.

### 4.3. Strengths and Limitations

The profiling systems of drug prescribing are inevitably “local”: they depend on the regulations in place, the responsible actors, the health care system in general, the available drugs, and so on. The study findings therefore do not generalise easily. The BDA, with its emphasis on preferred drugs and generic prescription targets, however, is of interest to any health care system aiming at containing costs and increasing the quality of drug prescribing.

Our database is limited in that no diagnoses or other clinical characteristics of patients were included. Furthermore, the analyses focused on eight specialisation groups we selected somewhat informally on the basis of characteristics mentioned in the method-section. But further analyses of all the specialisation groups would complete the results.

Our data analyses focused on the three drugs that are most notorious for preventing target achievement. We thus identified some mechanisms leading to missing/meeting targets. These, however, represent only a small part of the complexities of prescribing in practice.

The dataset included prescriptions and DDD amounts of all SHI members. Hence, we analysed the complete data for Bavaria in 1/2018, which include the prescriptions of all practitioners treating SHI members in the ambulatory sector. Because we received aggregated data, we only cumulated prescribing data and had no information on the prescriptions of a single practice. Errors from processing the printed prescriptions in the pharmacy data centre are identified and corrected/might occur in rare cases.

## 5. Conclusions

Prescribers are encouraged to seek information that is not biased by commercial interest. Drug bulletins, guidelines, and information from the KV and insurance companies are available at a low or even no cost. Thus, we hypothesize, these sources of information are less attractive and sometimes more difficult to obtain than glossy magazines (“comics”) or presentations by paid opinion leaders, in some cases in combination with gifts or meals. However, further research is necessary to verify this hypothesis.

The profiling of drug prescribing “works” via several mechanisms: the provision of general information regarding drugs, including indication, prices, and alternatives, individual feedback and its consequences, among these the prospect of grave sanctions if targets are repeatedly missed, although this happens only in very rare cases.

For administrators choosing appropriate variables (behaviours) and targets for drug prescribing surveillance, we suggest the following:

1. Targets must be relevant, so they cover the frequently prescribed drugs of a specialisation group.

2. They need a convincing evidence base.

3. They must be justified for prescribers, patients, and the public.

4. They must be adapted to specialisation and perhaps even sub-specialisation groups, such as child vs. adult care.

5. They must be realistic; that is, most prescribers must be in a position to meet the targets. If this is not the case, additional measures are needed to encourage the desired behaviour.

6. There must be additional strategies of information, feedback, and remedial measures.

7. They must be updated regularly, as shown by our findings regarding biosimilars.

## Figures and Tables

**Table 1:** Specialisation groups considered for more detailed drug analyses.

Analysed Specialisation Groups
Orthopaedics
Paediatrics
General internists
General practitioners
Neurology/psychiatry
Internists with a focus on cardiology
Internists with a focus on gastroenterology
Internists with a focus on pneumology

**Table 2:** Three drug targets with the highest prescription volume per selected specialisation group.

Specialisation Group	Drug Targets with High Volume	Characterisation of the Target	DDD Volume	Drug Target Achievement
Orthopaedists	Anti-inflammatory drugs/antirheumatics *	Generic	3,673,056.94	107.91%
	Drugs for the treatment of bone diseases **	Generic	1,864,718.78	103.38%
	Analgesics (without strong opioids) ***	Generic	634,758.24	101.72%
Gastroenterologists	PPI		1,320,355.50	111.82%
	TNF-alpha inhibitors	Recommended drug	508,012.62	146.99%
	Drugs with an effect on the RAS system	Generic	366,334.52	100.85%
General internists	Drugs with an effect on the RAS system	Generic	3,738,192.80	100,42%
	Combined group cardiovascular system	Generic	2,885,445.07	99.91%
	PPI		2,148,875.00	106.59%
Cardiologists	Drugs with an effect on the RAS system	Generic	3,478,049.40	101.91%
	Combined group cardiovascular system	Generic	2,460,292.26	101.05%
	Lipid-lowering drugs	Generic	1,654,913.32	94.80%
Pneumologists	Drugs for obstructive respiratory disease	Generic	10,045,618.00	103.45%
	Corticosteroids systemic	Generic	614,152.03	103.51%
	PPI		254,028.00	120.10%
Neurologists/ psychiatrists	Antidepressants (incl. ADHS drugs and antidementia drugs)	Generic	24,755,047.47	101.23%
	Other psychotropic drugs	Generic	7,072,299.75	99.46%
	Antiepileptics	Generic	5,319,697.57	96.82%
Paediatrics	Anti-inflammatory drugs/antirheumatics *	Generic	2,098,457.85	101.97%
	Drugs for obstructive respiratory disease	Generic	2,061,194.71	99.61%
	Ophthalmologic	Generic	1,222,314.17	121.37%
General practitioners	Drugs with an effect on the RAS system	Generic	290,176,258.39	100.69%
	Combined group cardiovascular system	Generic	198,480,232.04	100.46%
	PPI		104,936,679.00	106.70%

* Anti-inflammatory drugs, for example, NSAIDs, methotrexate, sulfasalazine. ** Predominantly for the treatment of osteoporosis. *** Analgesics without strong opioids, for example, paracetamol, acetylsalicylic acid. For detailed information about the drugs included in the individual drug targets, check the Supplementary Materials (Table S1).

**Table 3:** The three drugs mostly preventing the achievement of drug targets.

Drug 1	DDD Volume	Drug 2	DDD Volume	Drug 3	DDD Volume
**General internists**					
Combined group cardiovascular system					
Nebivolol (generic)	8680.00	Nepresol^®^	8456.621	Carmen^®^	7600.00
**Cardiologists**					
Lipid-lowering drugs					
Ezetrol^®^	115,970.00	Inegy^®^	42,750.00	Atozet^®^	27,440.00
**Neurologists**					
Other psychotropic drugs					
Quilonum^®^	354,153.602	Fluanxol^®^	148,831.497	Tavor^®^	86,836.00
Antiepileptics					
Orfiril^®^	141,923.323	Ergenyl^®^	123,383.735	Briviact^®^	38,674.20
**Paediatrics**					
Drugs for obstructive respiratory disease					
Flutide^®^	82,400.00	Viani^®^	79,650.00	Atrovent^®^	36,218.708

For further specification of the drugs and their active pharmaceutical ingredient, have a look at Supplementary Materials Table S3.

## Data Availability

Trial registration was not necessary, and permission from the Bavarian Inspecting Authority of Data Security was obtained.

## Supplementary Materials

The following supporting information can be downloaded at: https://www.mdpi.com/article/10.3390/ijerph21091174/s1, Table S1: Characterisation and definition of drug targets; Table S2: Number and description of relevant drug targets within individual specialisation groups; Table S3: Further specification of drugs of the drug targets analysed in Table 3 in the publication; Table S4: Description and characterisation of drug targets missed by more than 10% for the specialisation groups examined.
