# Supplementary material for: Controlling Prescribing through “Preferred Drug” Targets—The Bavarian Experience
Source: Int J Environ Res Public Health. 2024 Sep 3;21(9):1174. doi: 10.3390/ijerph21091174 (PMC11431696; doi:10.3390/ijerph21091174)
Supplement: Supplementary file 1 [file ijerph-21-01174-s001.zip › ijerph-3153426-supplementary.pdf]

Titel: Controlling prescribing through “preferred drug” targets – the Bavarian experience

Journal: International Journal of Environmental Research and Public Health

Authors: Julia Gollnick, Nikoletta Lippert, Julia Muth, Franziska Hörbrand, Kerstin Behnke, Peter Killian, Maria Sebastiao, Thomas Kühlein, Nobert Donner-Banzhoff

Contact: Julia Gollnick

Philipps-Universität Marburg

Institut für Allgemeinmedizin, Präventive, und Rehabilitative Medizin

Karl-von-Frisch-Straße 4

35043 Marburg (Germany)

+49 (06421) 28-65120

Julia.gollnick@staff.uni-marburg.de

Table S1 – Characterisation and definition of drug targets

Individual drug target	Characterization of drug target	Affecting and regulated ATC codes
Analgesics (without strong opioids)	Generic	N02 without (N02AA01, N02AA03, N02AA05, N02AA55, N02AB02, N02AB03, N02AB07, N02AC03, N02AC06, N02AE01, N02AX06)
Antibiotics	Generic	J01
Antidiabetics (without insulin)	Generic	A10B
Antiepileptics	Generic	N03
Antimycotics systemic	Generic	D01B, J02A
Antiparkinsonian drugs	Generic	N04
Antiphlogistics/antirheumatics	Generic	M01
Strong opioids	Generic	N02AA01, N02AA03, N02AA05, N02AA55, N02AB02, N02AB03, N02AB07, N02AC03, N02AC06, N02AE01, N02AX06
Corticosteroids systemic	Generic	H02
Endocrine therapy	Generic	L02 (without L02AE)
Hormonal contraceptive	Generic	G02BB01, G03A

Combined group cardiovascular system	Generic	C02, C03, C07, C08
Lipid-lowering drugs	Generic	C10
Drugs for obstructive respiratory disease	Generic	R03
Drugs for the treatment of bone diseases	Generic	M05
Ophthalmics	Generic	S01
Psychoanaleptics (without ADHS)	Generic	N06 (without N06BA02, N06BA04, N06BA09, N06BA12, N06BA14)
ADHS therapy	Recommended drug	N06BA02, N06BA04, N06BA09, N06BA12, N06BA14
Psycholeptics	Generic	N05
Drugs with an effect on the RAS system	Generic	C09
Rhinologics with corticosteroids	Generic	R01AD
Sex hormones and modulators	Generic	G03 (without G03A)
Antiplatelet therapy	Generic	B01AC
Urologics	Generic	G04BC; G04BD, G04BX18, G04C; Cialis® 5mg
Other antianaemics	Recommended drug	B03XA

Anticoagulants	Recommended drug	B01AA03, B01AA04, B01AE07, B01AF01, B01AF02, B01AF03
DOACs	Recommended drug	B01AE07, B01AF01, B01AF02, B01AF03
GnRH analogues	Recommended drug	L02AE
Colony-stimulating factors	Recommended drug	L03AA
MS drugs	Recommended drug	L03AB07, L03AB08, L03AB13, L03AX13, L04AA23, L04AA27, L04AA31, L04AA34, L04AC01, N07XX09
TNF-alpha inhibitors	Recommended drug	L04AB
PPI		A02BC

The list has been modified in accordance with the target definition of the Bavarian KV.

For a detailed list of targets and further information, check the definition of the Bavarian KV:

https://www.kvb.de/fileadmin/kvb/V10/Mitglieder/Service/Rechtsquellen/S-Z/KVB-RQ-Wirkstoffvereinbarung-Definition-Ziele.pdf

Table S2 – Number and description of relevant drug targets within individual specialisation groups

Specialisation group	Number of relevant drug targets	Name of drug targets
Ophthalmology	2	Corticosteroids systemic
		Ophthalmics
Doctors practising psychotherapy	3	Psycholeptics
		Psychoanaleptics (without ADHS therapy)
		ADHS therapy
Dermatology	4	Antibiotics
		Antimycotics systemic
		Corticosteroids systemic
		Sex hormones and modulators
Child and youth psychiatrist	5	Analgesics (without string opioids)
		Antiepileptics
		Psychoanaleptics without ADHS therapy
		Psycholeptics
		ADHS therapy
Gynaecology	5	Antibiotics
		Endocrine drugs
		Hormonal contraceptive
		Sex hormones and modulators
		Urologics
Internist subspecialisation pneumology	7	Antibiotics
		Corticosteroids systemic
		Combined group cardiovascular system
		Drugs for obstructive respiratory disease
		Renin-angiotensin affecting drugs
		Rhinologics with corticosteroids
		PPI
Orthopaedics	7	Analgesics (without strong opioids)

		Antiphlogistics/antirheumatics
		Corticosteroids systemic
		Drugs for the treatment of bone diseases
		TNF-alpha inhibitors
		DOACs
		PPI
Internist subspecialisation endocrinology	7	Antidiabetics (without insulin)
		Corticosteroids systemic
		Combined group cardiovascular system
		Lipid-lowering drugs
		Drugs with an effect on the RAS system
		Sex hormones and modulators
		PPI
Internist subspecialisation cardiology	7	Combined group cardiovascular system
		Lipid-lowering drugs
		Drugs with an effect on the RAS system
		Antiplatelet therapy
		Anticoagulants
		DOACs
		PPI
Internist subspecialisation rheumatology	7	Analgesics (without strong opioids)
		Antiphlogistics/antirheumatics
		Corticosteroids systemic
		Drugs for the treatment of bone diseases
		Drugs with an effect on the RAS system
		TNF-alpha inhibitors
		PPI
Urology	7	Antibiotics
		Strong opioids
		Endocrine drugs
		Drugs for the treatment of bone diseases
		Sex hormones and modulators
		Urologics

		GnRH analogues
Internist subspecialisation gastroenterology	8	Analgesics (without strong opioids)
		Antidiabetics (without insulin)
		Strong opioids
		Combined group cardiovascular system
		Lipid-lowering drugs
		Drugs with an effect on the RAS system
		TNF-alpha inhibitors
		PPI
Paediatrics	8	Analgesics (without strong opioids)
		Antibiotics
		Antiepileptics
		Antiphlogistics/antirheumatics
		Drugs for obstructive respiratory disease
		Ophthalmics
		Psychoanaleptics (without ADHS)
		ADHS therapy
Neurology	10	Analgesics (without strong opioids)
		Antiepileptics
		Antiparkinsonian drugs
		Antiphlogistics/antirheumatics
		Strong opioids
		Corticosteroids systemic
		Psychoanaleptics (without ADHS)
		Psycholeptics
		ADHS therapy
		MS drugs
Internist subspecialisation oncology	11	Analgesics (without strong opioids)
		Antibiotics
		Antimycotics systemic
		Strong opioids
		Corticosteroids systemic
		Endocrine drugs

		Drugs for the treatment of bone diseases
		Drugs with an effect on the RAS system
		Antianaemics
		Colony-stimulating factors
		PPI
Primary care	23	Analgesics (without strong opioids)
		Antibiotics
		Antidiabetics (without insulin)
		Antiepileptics
		Antiphlogistics/antirheumatics
		Strong opioids
		Corticosteroids systemic
		Combined group cardiovascular system
		Lipid-lowering drugs
		Drugs for obstructive respiratory disease
		Drugs for treatment of bone diseases
		Ophthalmics
		Psychoanaleptics without ADHS therapy
		Psycholeptics
		Drugs with effect on the RAS system
		Rhinologics with corticosteroids
		Sex hormones and modulators
		Antiplatelet therapy
		Urologics
		ADHS therapy
		Anticoagulants
		DOACs
		PPI
Anaesthesiology		
Surgery		
Oral and maxillofacial surgery	No data available	
Otorhinolaryngologist		
Internists without subspecialisation		

Internists with nephrology subspecialisation	
Internists with angiology subspecialisation	
Neurosurgery	
Specialists in rehabilitative and physical therapy	

The following table S3 includes further information about the drugs listed in table 4 of the publication. The active pharmaceutical ingredients of the named drugs are shown. These drugs prevent drug target achievement.

Table S3 – Further specification of drugs of the drug targets analysed in table 3 in the publication

**Further specification of the drugs**	
**Drug**	**Active pharmaceutical ingredient**
~~**Exforge® HCT**~~	~~Amlodipine, valsartan, hydrochlorothiazide~~
~~Preterax®/Bipreterax®~~	~~Perindopril/perindopril, indapamide~~
~~Dafiro® HCT~~	~~Amlodipine, valsartan, hydrochlorothiazide~~
Nebivolol (generic)	Nebivolol
Nepresol®	Dihydralazine
Carmen®	Lercanidipine
Ezetrol®	Ezetimibe
Inegy®	Ezetimibe, simvastatin
Atozet®	Atorvastatin, ezetimibe
Quilonum®	Lithium
Fluanxol®	Dlupentixol
Tavor®	Lorazepam
Orfiril®	Valproate
Ergenyl®	Valproate
Briviact®	Brivaracetam
Flutide®	Fluticasone
Viani®	Fluticasone, salmeterol

Atrovent®	Ipratropium bromide

Table S4 provides information on whether drugs are generic, recommended, or a PPI target and how many DDDs are prescribed within this drug target by the whole specialization group.

Table S4 – Description and characterisation of drug targets missed by more than 10% for the specialisation groups examined

Specialisation group	Missed drug targets	Characterisation of the target	DDD volume of the whole specialisation group within this target	Target achievement
**Orthopaedists**	Antiparkinsonian drugs	Generic	557.67	64.66%
	DOACs	Recommended drug	16,704.32	55.78%
	Ophthalmics	Generic	2,726.75	85.76%
**Gastroenterologists**	MS drugs	Recommended drug	366.29	34.79%
	Other antianaemics	Recommended drug	802.46	36.29%
	Anticoagulants	Recommended drug	32,796.84	69.69%
**General internists**	MS drugs	Recommended drug	645.7	42.76%
	Anticoagulants	Recommended drug	341,732.96	68.93%
	Rhinologics with corticosteroids	Generic	11,893.26	78.67%
**Cardiologists**	Anticoagulants	Recommended drug	728,220.06	58.89%
	Rhinologics with corticosteroids	Generic	3,213.34	87.65%
	Antidiabetics (without insulin)	Generic	227,441.22	87.68%
**Pneumologists**	Anticoagulants	Recommended drug	32,005.96	35.71%
	Ophthalmics	Generic	3,443.79	61.61%
	Antiparkinsonian drugs	Generic	2,092.67	81.85%
	Antiepileptics	Generic	2,879.33	89.93%

**Neurologists/ psychiatrists**	Anticoagulants	Recommended drug	7,990.90	16.58%
	Urologics*	Generic	51,366.27	85.14%
	DOACs	Recommended drug	7,288.90	89.54%
**Paediatricians**	Colony-stimulating factors	Recommended drug	151.30	27.97%
	TNF-alpha inhibitors	Recommended drug	22,932.42	35.81%
	MS drugs	Recommended drug	323.96	39.34%
	Other psychotropic drugs	Generic	52,183.11	62.62%
	Other antianaemics	Recommended drug	3,126.44	66.89%
	Antiparkinsonian drugs	Generic	2,084.84	78.17%
	Lipid-lowering drugs	Generic	8,141.90	79.35%
	Combined group cardiovascular system	Generic	64,311.94	84.07%
**General practitioners**	Anticoagulants	Recommended drug	22,223,847.10	71.54%
	Other antianaemics	Recommended	56,880.63	74.72%

* Mostly drugs for neurogenic bladder leader problems.
